# Rice Molecular Breeding Laboratories in the Genomics Era: Current Status and Future Considerations

**DOI:** 10.1155/2008/524847

**Published:** 2008-05-28

**Authors:** Bert C. Y. Collard, Casiana M. Vera Cruz, Kenneth L. McNally, Parminder S. Virk, David J. Mackill

**Affiliations:** ^1^Hermitage Research Station, Queensland Department of Primary Industries & Fisheries, 604 Yangan Road, Warwick, Queensland 4370, Australia; ^2^International Rice Research Institute, DAPO Box 7777, Metro Manila, Philippines

## Abstract

Using DNA markers in plant breeding with marker-assisted selection (MAS) could greatly improve the precision and efficiency of selection, leading to the accelerated development of new crop varieties. The numerous examples of MAS in rice have prompted many breeding institutes to establish molecular breeding labs. The last decade has produced an enormous amount of genomics research in rice, including the identification of thousands of QTLs for agronomically important traits, the generation of large amounts of gene expression data, and cloning and characterization of new genes, including the detection of single nucleotide polymorphisms. The pinnacle of genomics research has been the completion and annotation of genome sequences for *indica* and *japonica* rice. This information—coupled with the development of new genotyping methodologies and platforms, and the development of bioinformatics databases and software tools—provides even more exciting opportunities for rice molecular breeding in the 21st century. However, the great challenge for molecular breeders is to apply genomics data in actual breeding programs. Here, we review the current status of MAS in rice, current genomics projects and promising new genotyping methodologies, and evaluate the probable impact of genomics research. We also identify critical research areas to “bridge the application gap” between QTL identification and applied breeding that need to be addressed to realize the full potential of MAS, and propose ideas and guidelines for establishing rice molecular breeding labs in the postgenome sequence era to integrate molecular breeding within the context of overall rice breeding and research programs.

## 1. INTRODUCTION

Rice (*Oryza sativa*) is the well-known holder of two important titles: the most important food crop in the world and a model cereal species. Rice is the staple food in many parts of the world, including many developing countries in Asia, Africa, and Latin America. The projected increase in global population to 9 billion by 2050 and predicted increases in water scarcity, decreases in arable land, the constant battle against new emerging pathogens and pests, and possible adverse effects from climate change will present great challenges for rice breeders and agricultural scientists [1–4]. Because of rice’s global importance, small genome size, and genetic relatedness to other major cereals, efforts were undertaken to sequence the entire genomes of the two subspecies of rice—*indica* and *japonica*. Genome sequence drafts were completed for both subspecies in 2002 [5, 6] and a high-quality and annotated version of the *japonica* species was completed in 2005 [7], which represent landmark achievements in biological research.

One practical output from genomics research was the development of DNA markers (or molecular markers) in the late 1980s and 1990s. Marker-assisted selection (MAS)—in which DNA markers are used to infer phenotypic or genotypic data for breeding material—is widely accepted to have great potential to improve the efficiency and precision of conventional plant breeding, which may ultimately lead to the accelerated release of new crop varieties [8–13]. The potential advantages of molecular breeding demonstrated by numerous examples of MAS in rice and other crops have prompted many rice breeding and research institutes to establish biotechnology or DNA marker labs within the institute.

Genomics is the study of gene location, function, and expression. Strictly speaking, the study of gene location might be classified as molecular genetics research. However, for simplicity, we broadly define genomics as the study of genes and genomes, which includes identifying the location of genes as well as the study of gene function and regulation (expression). The beginning of the 21st century has been considered the dawn of the genomics era due to the enormous amount of genomics research in bacterial, plant, and animal species, as well as the rapid development of high-throughput equipment for whole-genome genotyping, gene expression, and genome characterization, and the establishment of advanced bioinformatics tools and databases. These rapid developments have irreversibly influenced and redefined plant breeding in the 21st century as “molecular plant breeding” or “genomics-assisted breeding” [14].

However, plant breeders and agricultural scientists face many challenges to integrate and exploit these new molecular and genomics-related technologies for more rapid and efficient variety development [15, 16]. In this article, we review the current global rice molecular breeding lab with an emphasis on recent research and the impact of rice genomics resources. We also review some current genomics research and promising new genotyping methodologies with high potential for applied outcomes. Finally, we consider the obstacles to the successful application of molecular genetics and genomics research in rice breeding programs and propose ideas on how some of these problems should be solved.

## 2. THE RICE MOLECULAR BREEDING LAB

### 2.1. View of the rice “pregenome sequence” molecular breeding lab

We arbitrarily define the “pregenome sequence molecular breeding lab” as before 2000. Although the first rice genome sequence drafts were published in 2002 and the complete genome sequence was published in 2005, sequence data were available before these publication dates so it is very difficult to exactly pinpoint the time when rice genome sequence data influenced applied rice genetics and breeding. In the early to mid-1990s, restriction fragment length polymorphism (RFLP) and random amplified polymorphic (RAPD) markers were commonly used for rice breeding research [17–21]. In Japan, RFLPs continue to be a marker system of choice [22]. Often, RFLP and RAPD markers were converted into second generation, polymerase chain reaction (PCR)-based markers called sequence tagged site (STS) markers to improve technical simplicity and reliability [23–25]. Simple sequence repeats (SSR; or “microsatellites”) became the most widely used markers in cereals and rice is no exception [26–28]. In earlier reports, the principles and techniques of detecting SSR polymorphisms were called simple sequence length polymorphism (SSLP) markers [28, 29]. SSRs are highly reliable (i.e., reproducible), codominant in inheritance, highly polymorphic (compared to other markers), and generally transferable between mapping populations. The only disadvantages of SSRs are that they typically require polyacrylamide gel electrophoresis and generally give information only about a single locus per assay.

The first SSRs were reported in 1996 [30]. By 1997, there were 121 validated SSRs, which were adequate for marker-assisted evaluation of germplasm and the construction of framework linkage maps but had limited use for MAS, due to limited genome coverage [29]. By 2001, there were a total of ∼500 SSRs that were developed from 57.8 Mb of publicly available rice genome data [31], which further increased the utility of these markers.

### 2.2. The postgenome sequence rice molecular breeding lab: opening the “treasure chest” of new rice markers

#### 2.2.1. SSRs

Analysis of the completed rice genome sequence provided the identification of literally tens of thousands of new targets for DNA markers, especially SSRs. Using publicly available BAC and PAC clones, more than 2200 validated SSRs were released in 2002 [32]. This was soon followed by 18828 Class I (di-, tri-, tetra-repeats) SSRs that were released after the completion of the Nipponbare genome sequence in 2005 [7]. This number is by far the largest number of publicly available SSRs for any crop species. The extremely high density of SSRs (approx. 51 SSRs per Mb) will provide a considerable “tool kit” for map construction and MAS for numerous applications. Given that many labs are currently well equipped for SSR analysis, it is highly likely that SSRs will continue to be the marker of choice for years to come.

#### 2.2.2. Single nucleotide polymorphisms (SNPs)

SNPs are the most abundant and ubiquitous type of polymorphisms in all organisms, and many researchers propose that these markers will be the marker of choice in the future [33]. In rice, SNPs can be readily identified by direct comparisons of Nipponbare and 93-11 genomes, or by sequence alignment with one or both reference sequences with available sequence data in public databases [34–36]. Recently, more SNP data have become available that have been generated by comparing partial sequences from multiple genotypes [37–39]. In some cases, DNA sequencing of target regions in specific genotypes is required. However, experimental validation of SNP-based markers is required since inaccuracies in sequence data have been reported [34, 36]. The ease with which SNPs can be identified in silico and increase in publicly available rice DNA sequence data will undoubtedly ensure that SNP-based markers will be more commonly used in the future.

It should be noted that lower levels of SNP marker polymorphism are usually detected in more closely related genotypes, which are more representative of breeders’ elite germplasm (*indica* × *indica* or *japonica* × *japonica*-derived material), when compared with the *japonica-indica* reference genotypes used to determine SNP frequency. The frequency of SNPs between subspecies was reported to be from 0.68% to 0.70%, whereas it was 0.03% to 0.05% between *japonica* cultivars and 0.49% between *indic* a cultivars [35]. Interestingly, SNPs were not evenly distributed along chromosomes.

#### 2.2.3. Indels

Insertion/deletion (indel) mutations are abundant mutations that occur in coding and noncoding regions. Indels can also be quickly identified in silico by direct comparions of *japonica* and *indica* genome sequences. The enormous number of indels between the two subspecies will provide an indispensable resource of polymorphic markers for *indica* × *japonica* populations or populations with specific introgressions [34, 35]. Either of the two rice genome reference sequences can be easily compared with other sequences for further indel identification, as was done between Nipponbare and Kasalath, a commonly used *indica* accession [40]. Like SNP-based markers, indels also need to be experimentally validated.

Introns are noncoding regions within genes and hence they “tolerate” insertion/deletion mutations compared with exons. Consequently, many indels have been identified in introns and these size polymorphisms have been exploited by the development of a new class of intron length polymorphic (ILP) markers [41]. Experimental validation of these markers indicated that the majority were reliable and codominant, and that although ILPs were designed from *indica/japonica* comparisons, they were also polymorphic between varieties within both subspecies although the level of polymorphism was lower.

#### 2.2.4. “Custom-made” markers

**Figure 1:**
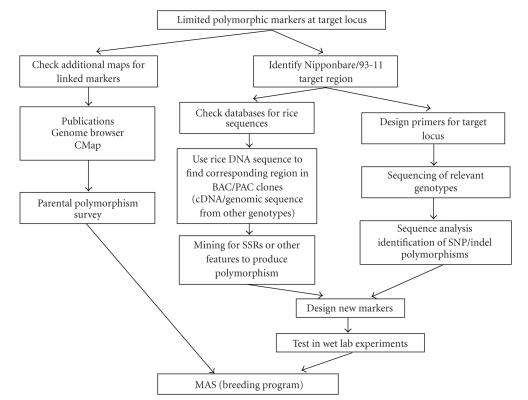
Process for developing custom-made rice DNA markers.

The great resource for molecular breeders is the DNA sequence provided by the genome sequences since it permits markers that are tightly linked to target loci to be “custom-made” or “tailor-made” to suit the aims of MAS. The large number of custom-made markers that have already been designed or the potential for new ones to be designed is a unique feature of the rice molecular breeding lab. The number of markers that can potentially be generated using the rice genome sequence in silico is practically unlimited (Figure 1). The markers might be derived directly from the Nipponbare/93-11 sequences or used to identify corresponding EST or genomic sequences available from databases (i.e., BAC or PAC clones containing target genes that may not actually be present in reference genotypes) [42–44]. In principle, custom-made markers can be any type although they most commonly include new SSRs, indels, PCR-based SNPs, and cleaved amplified polymorphic site (CAPS) markers—which are the technically the simplest markers to be used for marker genotyping [43, 45]. It should be noted that these markers must be tested in wet-lab experiments.

Candidate gene (CG) identification can be integrated with customized marker design and development. The advantage of CG-derived markers is that they are usually more tightly linked to the gene or QTL controlling the trait. This approach has been successfully used for identifying CGs associated with disease resistance, since cloned plant disease resistance genes possess conserved domains [46, 47].

### 2.3. Protocols, resources, and laboratory organization

Since marker genotyping methods were first developed in the 1980s, numerous protocols and variations now exist. Many protocols have been refined and optimized specifically for the lab in which marker genotyping is conducted and will depend on budget, equipment, and personnel. One feature of rice molecular breeding labs is their diversity. Molecular breeding labs require a large initial capital investment and since many labs are based in developing countries, the equipment and resources often differ markedly from those of well-funded labs in developed countries. The cost of marker genotyping is, therefore, a critical factor for the extent of MAS in rice, and this is likely to continue to be the case for years to come given the unlikely dramatic decrease in costs.

#### 2.3.1. DNA extraction protocols

Many general DNA extraction methods that are used in diverse plant species have been used in rice, from which it is relatively easy to extract DNA (see, e.g., [48–50]). Some methods have been specifically developed for rice [51]. The DNA extraction component is often the most time-consuming and laborious step of marker genotyping. For this reason, high-throughput methods using 96-well PCR plates have been developed [52]. The method by Xu et al. [52] does not require liquid nitrogen or freeze drying for initial grinding of leaf tissue or the use of organic solvents.

Alternative “quick and dirty” methods for DNA extractions in rice were evaluated and optimized at IRRI [53]. These methods were selected from published papers in the literature based on the time and resources required for using the protocols, as well as cost, and optimized for routine use. Two methods were selected as being the best when considering success of PCR amplification of SSRs, time, and cost [51, 54]. The modified method by Wang et al. [54] greatly reduced the time and cost for routine DNA extractions and was adapted into a 96-well plate method.

#### 2.3.2. SSR genotyping

SSR genotyping typically requires high-resolution electrophoresis, which is performed using polyacrylamide gels or, in some cases, high-resolution agarose. The majority of labs use standard gel electrophoresis equipment and stain gels with DNA-binding stains such as ethidium bromide, safer analogs, or silver staining (for acrylamide gels only). Multiplexing refers to the combination of primer pairs in PCR (multiplex PCR) or samples during gel electrophoresis (multiplex gel loading) [55]. This has considerable potential for increasing the efficiency of marker genotyping due to savings in time and resources. Multiplex loading is simpler, since there are fewer variables and it has been successfully demonstrated to greatly increase genotyping efficiency [23, 28, 29].

In some labs, capillary electrophoresis systems have been established. The accuracy of marker allele determination is one of the major advantages of these platforms, since size differences of 1 bp can be discerned. Multiplex loading can also be relatively easily performed using these genotyping platforms, which use fluorescently-labeled primers in PCR [56, 57]. These platforms can also be used for DNA sequencing, highlighting their versatility. Unfortunately, the cost of consumables, the initial expense of capital equipment purchase, and possibly the reliable acquisition of consumables and technical servicing may restrict their wider-scale adoption in actual breeding stations.

#### 2.3.3. SNP genotyping

The two simplest and most widely used methods for detecting SNP markers are PCR-based SNPs (that target SNPs by primer design) and restriction digestion of PCR amplicons, which are called cleaved amplified polymorphic site (CAPS) markers [43–45]. Komori and Nitta also used a variant of the CAPS method called derived CAPS (dCAPS), in which artificial restriction digestion sites are created in PCR amplicons. All methods use standard lab equipment [58].

Capillary electrophoresis platforms can also be used for SNP detection, based on the principle of single nucleotide primer extension (SNuPE; [35]). The high resolution of capillary electrophoresis equipment also permits small indels (say, <3 bp that are too small to be resolved on standard agarose or to be detected with acrylamide). A codominant single nucleotide length polymorphism marker (i.e., 1 bp indel) was developed from the intron region of the *Pi-ta* gene by Jiang et al. [59].

#### 2.3.4. Indel genotyping

One attractive feature of many indels, including ILPs, is that standard agarose electrophoresis or acrylamide gel electrophesis equipment and methods used for SSR detection can be used [41]. Another attractive feature of indels that are located within genic regions is that they are gene-specific markers, so the possibility of recombination between marker and gene is eliminated.

#### 2.3.5. Data management

It is important that molecular breeding labs have a system in place to store marker data, since they are an extremely useful resource for future breeding research. There is not a universal method for data storage—systems range from in-house Excel files to sophisticated laboratory information management systems (LIMS). We have found that standard database software is adequate for marker data storage. The development of template files and standard operating procedures for all researchers to use is more important. This information can be exploited for future genotyping activities. Careful data collation is essential to ensure that parental genotyping is not unnecessarily repeated and to determine opportunities for multiplexing.

**Figure 2:**
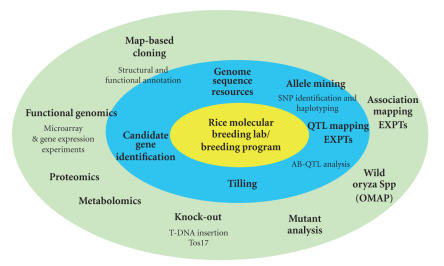
“Impact circle” overview of genomics and molecular research in rice. Research areas have been placed in either the inner or outer circle. Inner circle research activities are considered to provide more direct applied benefits (in terms of new markers, information for the development of new markers, or new breeding lines) to the rice molecular breeding lab/breeding program, which is located in the center. Research areas indicated in the outer circle are generally considered to provide indirect benefits to the rice molecular breeding lab/breeding program.

For labs that generate large amounts of genotypic data, a more formal LIMS could be appropriated (see Figure 2); some of these systems have been recently developed for general crop species [60, 61].

#### 2.3.6. Rice molecular breeding Internet resources

**Markers and maps**

The Internet has become a vital and convenient repository for marker and map data, and the rice molecular breeder must become familiar with these resources. There are excellent resources for published rice DNA markers that are maintained at the Gramene website [62, 63] http://www.gramene.org/ (these resources are the envy of other cereal researchers!). These web resources can be used for many applications, including obtaining SSR primer sequences, marker allele size data, and the map position of markers. Gel photos on a reference set of rice genotypes can also be obtained from this link. A large repository of published linkage maps, genes, QTLs, mutants, and references can also be searched in Gramene. The comparative map viewer (CMap) can be used to visually compare maps side by side [64].

The integrated rice genome explorer (INE; http://rgp.dna.affrc.go.jp/giot/INE.html) was developed to provide quick and simple correlations between genetic markers and EST, and physical maps with the rice genome sequence [65] are another excellent resource. These features can be viewed rapidly in the database.

**“Genome browsers”: the genome sequence resource for searching**

The completed rice genome sequence map would be of limited use if it was not easy to search. For this purpose, user-friendly “genome browsers” (Gbrowse) have been developed. The Institute for Genomics Research (TIGR) Gbrowse resource (http://www.tigr.org/tdb/e2k1/osa1/) was designed for scientists to data-mine the rice genome [66, 67]. The rice genome sequence has been organized into “pseudomolecules” which are virtual contigs of the 12 rice chromosomes. Each gene has been designated with a locus identifier that enables specific points of reference to be identified within the pseudomolecule. This resource consists of annotated genes, identified motifs/domains within the predicted genes, a rice repeat database, identified related sequences in other plant species, and identified syntenic sequences between rice and other cereals. The TIGR Gbrowse enables structural and functional annotations to be quickly viewed. The latest version of the rice genome browser supports “tracks,” which allow users to view specific features such as markers and putative genes within defined regions. Enhanced data access is available through web interfaces, FTP downloads, and a data extractor tool [68].

More recently, a genome browser was established within Gramene that enables the Nipponbare genome sequence to be quickly searched. This sequence is linked to genetic linkage maps in the Gramene database. Genome browsers are extremely user-friendly resources for assisting with basic and applied research.

### 2.4. Marker-assisted selection (MAS) in rice

MAS is the process of using DNA markers to assist in the selection of plant breeding material [11, 12, 69, 70]. Collard and Mackill [8] described three fundamental advantages of MAS compared with conventional phenotypic screening.

- It is generally simpler than phenotypic screening, which could save time, effort, resources, and, for some traits, money. Furthermore, MAS screening is nondestructive.
- Selection can be carried out at any growth stage. Therefore, breeding lines can be screened as seedlings and undesirable plant genotypes can be quickly eliminated. This may be useful for many traits but especially for the traits that are expressed at specific developmental stages.
- Single plants can be selected and their precise genotype can be determined which permits early generation selection in breeding schemes. For most traits, homozygous and heterozygous plants cannot be identified by conventional phenotypic screening. Using conventional screening methods for many traits, single-plant selection is often unreliable due to environmental effects, which can be variable.

One of the most important ways in which these advantages can be exploited by breeding programs is the more precise and efficient development of breeding lines during frequently-used breeding methods such as backcrossing, bulk, and pedigree methods [9, 13]. Target genotypes can be more effectively selected, which may enable certain traits to be “fast-tracked,” potentially, resulting in quicker variety release. Markers can also be used as a replacement for phenotyping, which allows selection in off-season nurseries, making it more cost effective to grow more generations per year or to reduce the number of breeding lines that need to be tested, by the elimination of undesirable lines at early generations [13]. MAS has numerous applications in rice (Table 1). Some MAS applications represent activities that are impossible using conventional breeding methods (e.g., marker-assisted backcrossing and pyramiding). Collard and Mackill [8] emphasized the importance of exploiting the advantages of marker-assisted breeding over conventional breeding in order to maximize the impact on crop improvement.

#### 2.4.1. Genotype identity testing

DNA markers can be used to simply and quickly identify varieties—or confirm the identity of a varietal impostor. For simple F_1_ hybrids, codominant markers can be used to determine whether putative hybrids are genuine. Multiple F_1_s can also easily be screened and desirable genotypes can be selected.

Seed purity or intra-variety variation can easily be tested using markers. This can be more accurate than phenotypic evaluation [71]. For the testing of hybrid rice lines, using STS and SSR markers was considerably easier than using typical “grow-out tests” that involve growing plants to maturity and evaluating purity based on morphological and floral characteristics [70, 72]. SSRs from mitochondrial genes have been targeted for the development of markers to study maternally inherited traits such as cytoplasmic male sterility or the maternal origin of rice accessions [73]. It has often been determined that relatively few well-chosen markers can provide sufficient data for varietal discrimination.

#### 2.4.2. Genetic diversity analysis of breeding material

There have been numerous research papers on the assessment of genetic diversity in specific germplasm collections using different types of markers [74, 75]. However, in recent years, SSRs have become the marker of choice for this application (see Table 1). An example was the use of SSR markers to broaden the genetic base of U.S. rice varieties [76]. DNA markers have also been used in hybrid rice breeding in order to predict genotypes that combine to give superior hybrid vigor [77].

#### 2.4.3. Gene surveys in parental material

The accurate evaluation of genes in breeders' germplasm is of great importance for the selection of parental lines and development of new breeding populations. Having gene information for specific target loci (deduced from markers) can be extremely useful for breeders to efficiently use germplasm. An example of this was demonstrated by Wang et al. [80, 114], who used a set of dominant allele-specific markers for surveying markers to detect the presence of the *Pi-ta* resistance gene for rice blast in a large germplasm collection (*n* = 141).

#### 2.4.4. Marker-evaluated selection (MES)

This novel approach was used to identify genomic regions under selection (i.e., allelic shifts) of breeding populations using a modified bulk-population breeding system in target environments [99]. This approach makes no prior assumptions about traits for selection; however, selection is imposed in target environments. High-density or whole-genome marker coverage is an important prerequisite for MES. Theoretically, once specific alleles or genomic regions have been identified to be under selection, they can be combined via MAS to develop new breeding lines that are the “ideotypes” (i.e., ideal genotypes).

#### 2.4.5. Marker-assisted backcrossing (MABC)

MABC is the process of using markers to select for target loci, minimize the length of the donor segment containing a target locus, and/or accelerate the recovery of the recurrent parent genome during backcrossing [115, 116]. These three levels of selection have been referred to as foreground, recombinant, and background selection, respectively [8]. Terms were described after Hospital and Charcosset [116], who referred to foreground selection as the selection of a target locus and background selection as the selection of the recurrent parent genome using markers on noncarrier chromosomes and also on the carrier chromosome. MABC is superior to conventional backcrossing in precision and efficiency. Background selection can greatly accelerate a backcrossing program compared to using conventional backcrossing [117]. Furthermore, recombinant selection can minimize the size of the donor chromosome segment, thus reducing “linkage drag”—a “universal enemy” of the plant breeder [115]. This approach has been widely used and, due to the prevalence of several rice “mega varieties,” it is likely to continue being a successful approach [118].

For basic research applications, the MABC approach can be used to develop near-isogenic lines (NILs) with far greater precision than conventional backcrossing. Near-isogenic lines are valuable tools to characterize individual genes or QTLs. However, in many situations, NILs produced, using conventional backcrossing possess, many unknown donor introgressions on noncarrier chromosomes (i.e., chromosomes without target genes) and large donor chromosomal segments on the carrier chromosome. By using an MABC approach, NILs could be developed to ensure that lines are not influenced by “background” donor introgression and possess minimal donor segments flanking the target locus. We propose that NILs developed using such approaches are referred to as “precision introgression lines” (PILs). Ideally, markers with known map or physical positions should be used for PIL development.

#### 2.4.6. Pyramiding

Pyramiding is the process of combining genes or QTLs in progeny usually arising from different parents [101, 119]. Using conventional methods, this is extremely difficult or impossible to do in early generations (e.g., F_2_ or F_3_) because single plants need to be screened for multiple diseases or pathogen races. Because of the importance of blast and bacterial blight, many pyramiding efforts have been directed toward breeding for resistance to these two diseases (Table 1). There is strong evidence that combining resistance genes may provide broad-spectrum resistance [88, 120–123].

Although widely used for combining disease resistance genes or QTLs, pyramiding can be used for other abiotic stress tolerance and agronomic traits. An example of pyramiding agronomic genes was the combination of three thermosensitive genetic male sterility genes [106].

#### 2.4.7. Using transgenes

There has been much research in developing transgenic rice lines for basic and applied research applications [124]. MAS is traditionally used to screen for transformants for the transgene(s) [111]. However, with the availability of transgenics in rice for several useful traits such as resistan ce to diseases (bacterial blight, blast, sheath blight, yellow mottle virus), resistan ce to insects (stem borer, leaffolders), resistan ce to herbicide, tolerance of abiotic stress (drought, salt), nutritional traits (iron and pro-vitamin A), and photosynthetic traits [125, 126]; there is a strong interest in using transgenes in breeding. Rice breeders are excited to transfer them to successful mega varieties through conventional backcrossing or MABC. For example, transgenic rice (southern U.S. japonica-type varieties) with inherent ability to produce beta-carotene developed by Syngenta is available at IRRI and in several other national programs. However, these cultivars are not adapted to the tropical conditions in Asia, where most consumers prefer indica-type rice varieties. Therefore, at IRRI, we are introgressing the beta-carotene loci from japonica-type donor varieties into popular indica-type Asian rice varieties, using MABC. Initially, we used 3 GR1 events (GR1-146, GR1-309, and GR1-652) as donor parents, while 2 IRRI-bred mega varieties (IR64 and IR36) and a popular Bangladeshi variety (BR29) were used as recurrent parents. Subsequently, we received 6 GR2 events (GR2-E, GR2-G, GR2-L, GR2-R, GR2-T, and GR2-W). Four indica varieties, IR64, IR36, BR29, and PSB Rc 82, were used as recurrent parents. Advanced backcross progenies are available and some are ready for field testing [127–129].

## 3. CURRENT GENOMICS RESEARCH AND PROMISING NEW GENOTYPING METHODS

Attendance at the most recent international rice genetics conference held in Manila, Philippines (2005), indicated a mind-boggling amount of current research activities in rice genetics and genomics. These developments have been outlined in general and specific review articles (see, e.g., the excellent reviews [14, 121, 130–134]). In this section, we provide a brief overview of some of these research areas, with a focus on selected current genomics research projects that in our opinion are directed toward tangible applied molecular breeding outcomes. We also review some potentially useful and recently developed genotyping methods that could be used in breeding programs.

### 3.1. A brief overview of recent rice functional genomics research and annotation of the rice genome

Although the DNA sequences for Nipponbare and 93-11 are complete, rice genome sequence resources are constantly being revised and updated in terms of gene annotation [67]. There are two levels of annotation: structural annotation which refers to gene identification based on ESTs and full-length cDNA (FL-cDNA) sequences, and functional annotation which refers to the determination of gene function [132, 135]. The generation of EST libraries and FL-cDNA libraries has occurred simultaneously with genome sequencing for both *japonica* and *indica* subspecies [136, 137].

Since the actual function of the vast majority of genes remains unknown, functional annotation relies primarily on bioinformatics evidence to assign gene function [138]. To systematically and efficiently annotate the rice genome, an automated system and database called rice genome automated annotation system (RiceGAAS) was developed. This system automatically searches for rice genome sequences from GenBank, and processes them based on gene prediction and homology search programs for structural annotation. To facilitate the efficient management and retrieval of data for rice genome annotation, annotation databases such as the rice annotation project database (RAP-DB) [139] were developed.

Research in plant functional genomics provides useful data for functional annotation [140]. Reverse genetics approaches (studying the effect of gene alterations on phenotype) such as generating specific gene knockouts by RNA interference (RNAi), transfer-DNA (T-DNA), and transposon-mediated (*Ac*, *Ds*, *Ac/Ds,* and *Tos17*), and chemical/irradiated mutants have been successfully used to elucidate gene functions and determine tissue- or organ-specific gene expression (by using reporter genes) [141–146]. There are literally hundreds of thousands of mutant lines, albeit only a very small number of genotypes produced by basic research labs around the world can be screened for specific genes. Data generated by reverse genetics studies are publicly available and have been stored in curated databases such as the International Rice Information System (IRIS) [147], OryzaGenesDB [148], and EU-OSTID [149] for greater dissemination to the wider scientific community.

Microarrays have been widely adopted by plant scientists to study gene function. In rice, microarrays have been used to study processes related to yield (e.g., grain filling) and response to biotic and abiotic stresses [150–154]. Many databases have been developed to store gene expression data (reviewed in [135]). Most microarray studies have used gene-specific probes to detect gene expression and, hence, new “tiling microarrays” may study whole-genome expression, which is more informative because it is less biased [155, 156].

Although, to date, progress has been limited in rice, proteomics research also offers great promise for determining gene functions [157, 158]. In the future, it is hoped that a complete integration with proteomics and metabolomics will provide the ultimate data to elucidate not only individual gene functions but also complex pathways [135].

The generation of a deluge of genomics data has been accompanied by several integrative bioinformatics tools and databases. One notable example is called “Rice PIPELINE” which was developed for the collection and compilation of genomics data, including genome sequences, full-length cDNAs, gene expression profiles, mutant lines, and *cis* elements from various databases [159]. Rice PIPELINE can be searched by clone sequence, clone name, GenBank accession number, or keyword. Another web-based database system, called “PlantQTL-GE,” was developed to facilitate quantitative traits locus (QTL)-based candidate gene identification and gene function analysis [160]. This database integrated marker data and gene expression data generated from microarray experiments and ESTs from rice and *Arabidopsis thaliana*. Specific QTL marker intervals or genomic regions can be targeted for candidate gene analysis, which could be useful for identifying new candidate genes. Both databases are publicly available.

### 3.2. Current applied genomics research highlights

#### 3.2.1. Association of candidate defense genes with quantitative resistance to rice blast: a case study

The candidate gene approach has been used to integrate the molecular analysis of host-pathogen interactions, gene mapping, and disease resistance in rice. Candidate genes are similar to known genes or conserved motifs that make it possible to infer their biological functions [161]. Through their association with disease resistance, they become candidate defense response (DR) genes [122, 162, 163]. Advanced backcross lines of Vandana × Moroberekan, a japonica cultivar from Africa exhibiting durable quantitative resistance to blast in Asia, were used to demonstrate this approach for blast resistance. To accumulate different genes with quantitative resistance to blast, 15 BC_3_F_5_ lines of Vandana × Moroberekan showing partial resistance at IRRI and Cavinti, Philippines, and carrying DR candidate alleles were selected and crossed in all pairwise combinations. Plant selections based on blast resistance and agronomic acceptability were made in F_2_ and F_3_ populations, and the top 60 F_5_ selections were evaluated in multilocation environments.

To identify DR candidate genes in the progenies, molecular analyses of rice genes involved in quantitative resistance were done in selected F_4_ lines, using STS markers derived from rice candidate gene sequences and SSR markers located in the region of each candidate gene BAC clone showing polymorphisms between Vandana, Moroberekan, and their progenies. A total of 11 candidate genes were identified based on converging evidence (i.e., mapping, phenotyping, selection, microarray analysis) and used in this study. These candidate genes with known biological functions were oxalate oxidase/germin-like proteins, aspartyl protease (Esi-18), 14-3-3 proteins, PR-1, PBZ (PR10A), rice peroxidase (POX 22.3), heat shock protein (HSP90), putative 2-dehydro-3-deoxyphosphoheptonate aldolase, thaumatin-like pathogenesis-related protein, glyoxylase 1 (*Oryza sativa*), and S-adenosyl L-homocystein hydrolase. DR candidate genes were examined using in silico analysis of their sequences retrieved from the Rice Genome Program database. For genes occurring in gene families such as oxalate oxidase belonging to germin-like proteins, phylogenetic trees using the retrieved sequences were constructed to determine their relatedness and groups. The conserved promoter motifs were also compared and *cis*-elements in the 1000-bp upstream regions were identified. For each gene, there was variation in the copy number of *cis*-elements related to biotic stress responses, such as W box, WNPR1, and WRKY. This study suggested that these genes have potential associations with the response of rice to pathogen infection such as the blast fungus *Magnaporthe oryzae*.

#### 3.2.2. Identification of SNP by Eco-TILLING at specific candidate genes

TILLING or “targeting induced local lesions in genomes” is a reverse genetics technique developed to identify variation in *Arabidopsis* mutant libraries obtained from chemical mutagenesis with EMS [164, 165]. The approach involves creating pools of mutant lines followed by amplification with differentially labeled, locus-specific primers on these pools. If a pool contains a mutant variant, then denaturation/renaturation of the PCR products will allow heteroduplex mismatch molecules to be formed. Treatment of the products with the single-strand-specific endonuclease CEL1 will cleave a mismatch site and generate fragments that on separation and visualization by fluorescence will indicate the position of the mutation in the amplicon. Eco-TILLING is the application of this technique to discover allelic variation in natural populations. TILLING is accomplished using pools of mutant library lines having a majority of the wild-type allele at a given locus while Eco-TILLING contrasts a reference line, such as the source of the sequence with a single diverse germplasm accession. The main requirement for both TILLING and Eco-TILLING is sufficient sequence information for the design of locus-specific primers. Hence, SNP discovery and genotyping can proceed without the need for de novo sequencing, a requirement of other SNP genotyping tools prior to assay design.

At IRRI, we have designed locus-specific primers for a range of candidate genes putatively involved in drought, general stress response, and grain quality is leveraging the high-quality sequence information for the japonica-type Nipponbare [7]. Candidate genes were identified using convergent information taking into account genome annotation, involvement of the ortholog in another species, expression data, and colocalization with QTLs. Candidate genes for drought include DREB2a, ERF3, sucrose synthase, actin depolymerizing factor, and trehalose-6-phosphate phosphatase, among others. We have conducted Eco-TILLING at these candidate genes using a diverse collection of 1536 *O. sativa* accessions from the international Genebank collection contrasted to both japonica-type Nippponbare and indica-type IR64. Depending on the contrast, from 4 to 9 haplotypes have been discovered in about 1 kb at the candidate gene locus. Representative types for the haplotype mismatch patterns have been sequenced, and association tests with phenotypic data for vegetative-stage drought characters are under way. We have also optimized a procedure that allows TILLING/Eco-TILLING products to be detected on agarose gels, thus eliminating the need for fluorescent labeling and the use of an automated genotyper, with savings in both time and costs [166]. This simplified procedure is now our method of choice and its application to breeding will be described later.

#### 3.2.3. Genome-wide SNP discovery in diverse rice germplasm

The availability of the high-quality sequence of Nipponbare provides the unprecedented opportunity for genome-wide SNP discovery and improving our knowledge about allelic diversity in rice. IRRI along with partners in the International Rice Functional Genomics Consortium has undertaken a project to identify genome-wide SNP in a diverse collection of 20 varieties [167] with funding from IRRI, the Generation Challenge Program, and USDA-CSREES. The diverse varieties include representatives from all variety groups—temperate and tropical japonica, aromatic, aus, deep-water, and indica types—with Nipponbare included as a control. The technology being used for SNP discovery is hybridization to very high-density oligomer arrays pioneered by Perlegen Sciences, Inc. (Mountain View, Ca 94043, USA). On these arrays, four 25-mer oligomer features are tiled for each of the strands, where the middle base is present as A, T, C, or G for the four features with a single base offset occurring before the next set of features. Hence, 8 oligomer features interrogate each base of the sequence of the target genome during hybridization. Application of Perlegen’s technology has led to the identification of large sets of SNPs for human [168], mouse [169], and *Arabidopsis* [170].

Funding was available for SNP discovery in 100 Mb of the rice genomes. Consequently, only the nonrepetitive regions of the Nipponbare genome were selected for tiling onto high-density oligomer arrays. However, the nonrepetitive regions span the entire genome with the majority of 100 kb windows containing several or more tiled regions. Following hybridization of the query genomes to arrays, about 260000 nonredundant SNPs were identified by Perlegen’s model-based algorithms. Efforts are ongoing to extend this collection by applying the machine-learning-based techniques developed for the analysis of the Arabidopsis project [170].

The set of Perlegen model-based SNPs provides about 93% genome coverage by the criterion that at least 1 SNP occurs per 100 kb of the genome. Since existing estimates of linkage disequilibrium (LD) in rice indicate that LD extends to 100 kb or longer [171, 172], then the SNP dataset should be sufficient for identifying a collection of tag SNPs that define haplotype blocks across the rice genome. This set of tag SNPs can then be used to undertake whole-genome scans in a wider collection of rice varieties, with the resulting genotypic data applied to association studies with detailed phenotypes for traits of interest.

#### 3.2.4. Exploiting wild species

Landraces and wild species of rice (genus *Oryza*) possess an underused source of novel alleles that have great potential for crop improvement of cultivated rice species (*O. sativa* and *O. glaberrima*), since they possess new genes that could be exploited for yield increases and for developing resistance to biotic stresses and tolerance of abiotic stresses [173, 174]. Consequently, many experiments have attempted to use wild sources to develop new breeding material and also characterize genes and QTLs from these sources. The advanced backcross QTL analysis (AB-QTL) approach—which is a method for integrating QTL mapping with simultaneous line development—has been widely used to introgress wild genes and QTLs into adapted varieties with great success for agronomic traits and yield (reviewed in [174]).

Introgression lines (ILs) are derived by generating backcross lines using MAS with relatively large, different donor chromosomal segments from wild or exotic genotypes [119, 175]. ILs are useful for many applications in genetic analysis (e.g., high-resolution mapping of QTL regions), since phenotypic evaluation can be performed over multiple years and environments. In a study analyzing ILs developed from *Oryza rufipogon* in an *indica* background (Teqing), many putative QTLs for yield and yield components were detected [176].

Genome sequence research using wild species is well under way. The *Oryza* Map Alignment Project (OMAP) was initiated to construct physical maps (derived from BAC clones) of 11 wild and 1 cultivated species (*O. glaberrima*) and align them to the Nipponbare reference genome sequence [177, 178]. Advanced backcross populations (BC_4_F_2_) of 3 OMAP wild accessions are also being generated for mapping important traits. Apart from providing insights into evolution of the *Oryza* genus, other expected outcomes are the identification of new genes and QTLs that could be subsequently incorporated into adapted rice varieties.

#### 3.2.5. Association mapping

Despite the widespread use and success of QTL mapping for identifying QTLs that control traits, the method has inherent limitations [179, 180]. In practice, mapping populations are derived from bi-parental crosses that represent only a small fraction of the total allelic variation, and QTL mapping experiments may require a large investment in resources. Association mapping—based on linkage disequilibrium—may bypass these limitations of QTL mapping because a greater number of alleles are analyzed and historic phenotypic data for multiple traits can be readily used without the need for a specific evaluation of populations generated solely for the purposes of QTL mapping [181, 182]. Furthermore, association mapping can offer improvements in resolution because analysis is based on the accumulation of all meioses events throughout the breeding history.

Linkage disequilibrium has been estimated in rice to be approximately 100 to 250 kbp based on the characterization of two genes, *xa5* (chromosome 5) and *Waxy* (chromosome 6) [171, 172]. A more recent study indicated that the extent of LD was much larger: 20–30 cM [183]. The former estimate suggests that high-density whole-genome scans are required for efficient association mapping in rice. An alternative approach would be to focus on regions previously delimited by QTL analysis or regions in combination with candidate gene analysis.

Several recent studies have investigated “population structure” in rice, which is important for controlling the false discovery rate [83, 184, 185]. Various methods of data analysis have been evaluated. An example was the use of discriminant analysis involving markers associated with previous QTLs [186]. Discriminant analysis results were consistent with previous QTL results, although additional markers, not identified by QTL mapping methods, were detected which may indicate new loci associated with specific traits.

The “foundation” of previously identified QTLs for numerous traits, the availability of candidate genes from genomics research, and further improvements in statistical methodology [184] are likely to ensure that more rice researchers use association mapping approaches in the future.

### 3.3. Recent and new marker genotyping methods

#### 3.3.1. Optimizing and refining current protocols

One very important point we would like to emphasize before reviewing new technology is that there are great opportunities for further optimization of currently used protocols, especially in terms of cost and throughput. Furthermore, many innovations on standard methods are possible (see, e.g., [187]). This is important because many labs have already made a considerable investment in lab equipment and have the technical expertise to use specific protocols using specific markers.

As discussed earlier, multiplexing has considerable potential for increasing the efficiency of marker genotyping although this has not been extensively explored in rice. Multiplex PCR could be complicated since numerous variables (primer combination, annealing time and temperature, extension time and temperature, and concentrations of primers and magnesium chloride) are involved [188, 189]. However, in many cases, the investment in time and resources may be justified. Coburn et al. [57] reported 80% successful PCR amplification for duplex PCR. Multiplex loading is simpler and in our opinion could be applied on a much wider scale. At IRRI, loading of two or even three markers (A. Das, pers. comm.) is frequently possible, which saves time and resources. Of course, information regarding marker allele sizes is a prerequisite for multiplex loading.

#### 3.3.2. Considering the adoption of new genotyping methods

Many new promising genotyping methods could improve efficiency in terms of time and potential cost [190]. Most of these methods are targeted toward SNPs but most of them could be adapted for other marker types. Interestingly, there are many high-throughput SNP genotyping platforms (that have often been developed for medical applications), yet there has been no universally adopted system [191, 192].

In the context of plant breeding, there are several important considerations. Cost is critical due to the large number of samples breeders evaluate. Furthermore, 3 to 6 target traits usually segregate in a single population so the frequency of lines with all the desirable gene combinations is very low. This could undermine the suitability of some high-throughput whole-genome profiling programs, although there could be numerous applications in basic research.

Obviously, some genotyping methods will be more suitable for specific labs than others. For this reason, we have classified these methods into two groups: regional hub labs and remote breeding stations. A regional hub lab is defined as a research institute with a critical mass of scientists who receive sufficient funding for long-term, broad objective breeding research that includes genomics research (e.g., CGIAR centers and national breeding institutes). We refer to a remote breeding station as a “smaller” lab that has more limited capacity for marker genotyping in terms of funding and resources.

#### 3.3.3. Remote breeding station lab 1: gel-based methods

**PCR-based SNP methods**

PCR-based SNP detection methods that use standard agarose or acrylamide electrophoresis are obviously attractive because they are technically simple and no further investment in equipment is required. The simplest form of PCR-based SNP marker is based on designing PCR primers such that a forward or reverse primer has a specific dNTP at the 3′ end; PCR amplification is successful for the appropriate primer-template combination and fails when the specific 3′ base in the primer is not complementary to the template [43]. Reliability has been an important issue with designing PCR-based SNP markers; hence, several studies, exploring methods to improve reliability including the use of additional primers, have been conducted [193–195]. Hayashi et al. [43] introduced an artificial mismatch at the 3rd base from the 3′ end—in addition to the last 3′ base—which was found to increase specificity; a 67% success rate was found for 49 target SNPs. This method can be used to develop codominant allele-specific markers. Overall, these methods are useful to complement the arsenal of CAPS markers for whichtarget SNP-containing sites are not available.

**Heteroduplex cleavage SNP detection methods**

TILLING and Eco-TILLING methods (discussed previously) are reverse genetics methods used to identify SNPs in target genes in mutants and germplasm collections, respectively. However, simplified TILLING/Eco-TILLING methods, using standard polyacrylamide or agarose gel electrophoresis detection methods, could be applied for MAS and would be especially useful in situations, where it is difficult to find other types of polymorphic markers [166, 196]. This method relies on the principle that CEL I cleaves heteroduplexes at the position of SNPs.

In brief, the method involves the following steps.

- PCR amplification of the region of interest in parental lines (A and B) (homozygous).
- The PCR products are combined in equal concentration and subjected to CEL I digestion (TILLING/Eco-TILLING) in an agarose procedure to test for polymorphism.
- DNA is extracted from each member of the breeding population (RIL-homozygous) and quantified.
- DNA extracted from either of the parental lines (e.g., parent A) is combined with DNA from each of the RILs in a 1 : 1 ratio.
- The mix is subjected to CEL I digestion. If an SNP is detected, this indicates that the allele carried by the RIL is unlike that of the parent used to create the mix (in this case, parent A).

One possible limitation of this procedure is that it would be ideally done on homozygous lines. If there is doubt, the assay should be conducted with just the DNA from each of the RILs; no SNPs should be detected.

**PCR-RF-SSCP**

polymerase chain reaction- (PCR-) restriction fragment- (RF-) single-strand conformation polymorphism (SSCP)—abbreviated to PRS—is essentially based on a combination of the CAPS technique (i.e., restriction digestion of gene-specific PCR products) with SSCP, which on its own can be used for SNP detection of small PCR amplicons (100–400 bp) using polyacrylamide gel electrophoresis (PAGE) [197–199]. This method has been successfully used to detect SNPs in rice and other crops. One of the advantages of this method is that much longer PCR amplicons (>2000 bp) can be scanned for SNPs, and it may be well suited for labs with technical expertise in polyacrylamide gel electrophoresis and/or silver staining.

#### 3.3.4. Remote breeding station lab 2: non-gel-based methods

**Dot blots**

Dot blots have been used for genotyping of rice breeding material [200]. The main advantages of this methodare that gel electrophoresis and even PCR in some cases are not required. This method used cultivar-specific sequences that were previously identified by AFLP, STS, or PRS. Genomic DNA from rice samples was spotted on membranes and short oligonucelotide (28–45 bp) or digoxigenin (DIG)-labeled PCR products (102–466 bp) were used as probes. DIG labeling methods avoid the use of radioisotopes, which is preferable in most labs and very important for remote breeding stations due to delivery, storage, and disposal. Relatively high DNA yields were required for this method (3.5–5 *μ*g).

The dot blot genotyping method was later extended to a robust SNP detection [201]. In this method, two nucleotide probes (17 nt) were used: one allele-specific probe was DIG-labeled (at the 5′ end) and the other allele probe was unlabeled, following the principles of competitive allele-specific short oligonucleotide hybridization, which improves specificity. The probe targets were PCR products that contained the SNP regions. This method has potential for high-throughput capacity since 864 samples were blotted on a single membrane. Dot blot genotyping has been used for high-throughput, large-scale MAS in commercial companies [202]. Dot-blot assay was used in advanced Basmati-derived lines that have reached the replicated yield trial at IRRI’s breeding program (Reveche et al., unpublished data). This method, however, is not yet in routine use but offers great potential for MAS in breeding program.

#### 3.3.5. Regional hub lab

**Capillary electrophoresis platforms for SSR genotyping**

To maximize the efficiency of multiplexing using capillary electrophoresis platforms, marker “panels” can be assembled, which consist of markers with no overlapping allele size ranges or the same fluorescent dyes [56, 203]. In general, panels of any size and for any traits can be designed based on available primer resources and previously determined allele sizes. Coburn et al. [57] reported assembling panels consisting of 6 to 11 SSRs that were evenly spaced along all 12 chromosomes; most panels were designed such that they are chromosome-specific. A greater flexibility of panel design was demonstrated in maize, in which primers were redesigned for specific SSR loci from sequence data [114]. This permitted a tenplex level of multiplexing (i.e., scoring of 10 individual SSR marker alleles in a single gel lane). Although these panels from these two examples were designed for whole-genome scans, they have wider potential in routine MAS. Furthermore, generic fluorescently labeling primer methods, which greatly reduce costs, are other innovative methods by which the cost efficiency of capillary electrophoresis methods can be improved [204, 205]. In our opinion, it would also be feasible to adopt capillary electrophoresis systems in some remote breeding stations.

**SNuPE**

Many SNP detection methods are based on the commonly used principle of single nucleotide primer extension (also called single base extension, SBE). Briefly, this method works by using a genotyping primer that immediately precedes an SNP at the 3′ end in the template. This genotyping primer is extended with a specific fluorescently labeled dideoxy nucleotide (ddNTP) that is detected, which permits genotyping at a target locus. SNuPE can be performed using capillary electrophoresis systems, which could be very convenient if these platforms have been set up in labs for SSR genotyping. Capillary electrophoresis platforms have a very high throughput capacity: a pilot study in maize indicated that 1200 genotypes could be analyzed per day [206].

**FRET-based genotyping**

SNPs have become prominent in rice functional genomics research because of their advantage of being prevalent in the genome. For example, a recent study has reported an average occurrence of one SNP for every 40 kb in target regions in chromosomes 6 and 11 (S. McCouch, pers. comm.). If these SNPs are informative and exist in alternate alleles of a gene for resistance and susceptibility, for example, the *Xa21* gene for bacterial blight resistance, they would become useful candidates for marker development. At IRRI, we have adopted a method for SNP detection that uses the system known as fluorescence resonance energy transfer (FRET).

FRET is a radiation-less transmission of energy from a donor molecule to an acceptor molecule when they are in close proximity to one another (typically 10–100 Å). It has been mostly used in biomedical research and drug discovery to detect SNPs in the human genome [207, 208] and in homogeneous DNA diagnostics [209] as well as for other applications in protein interaction analysis [210]. In the conventional FRET reported by Takatsu et al. [211], the detection method requires special fluorescence-labeled probes, which are expensive and difficult to optimize. Later in the same year, Takatsu et al. [212] developed a method based on single base extension and applied SYBR Green I (bound to double-stranded DNA) as an energy donor and fluorescence-labeled ddNTP as an energy acceptor. This method avoids difficult probe design and allows a significant reduction in detection cost.

We have adapted the method for large-scale MAS in rice and further reduced the cost by optimization of expensive reagents (e.g., enzymes) during purification steps of single-stranded DNA prior to SBE. We employed the method as an SNP genotyping technique with the advantage of being high-throughput and non-gel-based. Here, the amplified genomic DNA containing the polymorphic site is incubated with a primer (designed to anneal immediately next to the polymorphic site) in the presence of DNA polymerase, SYBR Green I, and ddNTP labeled with a fluorophore (ROX or Cy5). The primer binds to the complementary site and is extended with a single ddNTP. When SYBR Green I is excited at its excitation wavelength of 495 nm, it will transfer the energy to the ddNTP at the polymorphic site next to it. High fluorescence intensity will be measured at each emission wavelength for SYBR Green I and the respective fluorophores for a resistant and susceptible allele, so SNP can be discriminated after the SBE reaction.

## Microarray-based genotyping (MBG)

**(A) SNP genotyping of alternate alleles**

DNA microarray technology provides a snapshot of gene expression levels of all genes in an organism in a single experiment. Depending on the objective of the experiment, it allows the identification of genes that are expressed in different cell types to learn how their expression levels change in different developmental stages or disease states and to identify the cellular processes in which they participate. This technology platform has also been used in genotyping studies, such as the tagged microarray marker (TAM) approach and the high-throughput system that makes genotyping efficient and low cost [213]. An alternative and simpler microarray technique was described by Ji et al. [214]. MBG is based on simple hybridization with fluorescence-labeled probes, which anneal with specific alleles in PCR products. MBG for MAS of specific genes needs printing of PCR products derived from breeding materials on glass. The alternate probes of the gene (e.g., *xa5* gene for bacterial blight resistance) are labeled with fluorophores, such as Alexa-Fluor 546 (or Cy3) for the R allele and Alexa-Fluor 647 (or Cy5) for the S allele. MBG is useful when the number of samples increases, thus decreasing the cost per data point. In designing an experiment for marker-assisted breeding, we can save time, space, and labor by establishing computer-aided data acquisition. MBG is one of the most advanced techniques for automated data processing.

Although the use of some expensive equipment, including the arrayer and scanner, may make users think twice, the cost per sample will be remarkably lower by using less expensive supplies and reagents that are commercially available.

**(B) Single-feature polymorphism (SFP)**

Microarray-based genotyping that used indel polymorphisms or SFP provides the means to simultaneously screen hundreds to thousands of markers per individual. This technology is particularly suited to applications requiring whole-genome coverage, and the relatively low cost of this assay allows a genotyping strategy using large populations. Along with foreground selection for the target traits, high-resolution whole-genome selection will provide a greater capacity for background selection to retain the positive attributes of popular varieties in backcrossing programs. Obtaining graphical genotypes of individuals will facilitate the pyramiding of desirable alleles at multiple loci and will shorten the time needed for developing new varieties.

SFP assays are done by labeling genomic DNA (target) and hybridizing it to arrayed oligonucleotide probes that are complementary to indel loci. The SFPs can be discovered through sequence alignments or by hybridization of genomic DNA with whole-genome microarrays. Each SFP is scored by the presence or absence of a hybridization signal with its corresponding oligonucleotide probe on the array. Both spotted oligonucleotides [215] and Affymetrix-type arrays [216] have been used in these assays. For genotyping large populations, the cost per individual is more critical than the cost per data point. Spotted oligonucleotide microarrays have the potential to provide low-cost genotyping platforms [217]. The availability of genomic sequences from multiple accessions presents opportunities for the design of spotted long oligonucleotide microarrays for low-cost/high-density genotyping of rice.

The SFP genotyping slide for rice has been developed in the laboratory of D. Galbraith, University of Arizona, Ariz, USA [218]. Using the publicly available genomic sequences of rice cultivars Nipponbare and 93-11 representing the *japonica* and *indica* subspecies, respectively, they made alignment of these sequences and identified 1264 SFPs suitable for probe design. With a median distance between markers of 128 kb, the SFPs are evenly distributed over the whole genome. An early result using these probes showed conservatively 30–50% polymorphism between a pair of rice lines (the lowest between *japonica* types). Thus, a single contrast produces around 400 well-spaced, polymorphic gene-based markers for any pair of unrelated parental lines. One advantage of the DNA hybridization-based genotyping procedure is that it can be used for quantitative genotyping of pooled samples.

Both of these microarray-based genotyping platforms can be combined for foreground (e.g., SNP genotyping of alternate alleles) and background selection (e.g., SFPs) in breeding programs.

**MALDI TOF MS**

Matrix-assisted laser desorption/ionization time-of-flight mass spectrometry (MALDI TOF MS) has been used for SNP genotyping in other crops such as barley and oilseed rape [219, 220]. The principle of mass spectroscopy is based on mass-to-charge ratio rather than electrophoretic mobility. SNP genotypes can be discriminated after SNuPE and then determining the molecular weight differences for the incorporated ddNTPs. This system has potential for high-throughput genotyping in regional hub labs because of the capacity to screen large numbers, speed of genotyping (seconds compared with hours for gel-based systems), amenability to automation, and low-cost potential.

## 4. CRITICAL ASSESSMENT OF THE IMPACT OF GENOMICS RESEARCH

### 4.1. Benefits to breeding

To date, the outcomes from genomics research have had three main benefits to breeders: increased knowledge regarding important traits, the generation of new breeding lines, and a vast array of DNA marker tools. Genomics research outcomes will provide considerably more information on the biology of traits, especially for complex quantitative traits for which information can be very limited [14]. Improved knowledge regarding complex traits can be extremely useful for breeders. Currently, there is an enormous amount of QTL and candidate gene data for these traits that will be continually refined and validated until specific genes are identified.

In applied terms, one important tangible benefit has been the generation of new breeding lines arising from QTL mapping experiments. These lines may include the “best” lines segregating for the traits under study. Numerous introgression lines or chromosome segment substitution lines (CSSL) and NILs developed for specific traits have considerable potential for breeding programs [221, 222]. As discussed earlier, many new breeding lines with wild donor introgression are the output from AB-QTL analysis experiments [174]. For breeding programs, ILs or AB-QTL analysis lines can be rapidly converted into NILs via an MABC approach using only a small number of backcrosses.

From the molecular breeding perspective, the most tangible benefit from genomics research is the wealth of DNA markers associated with traits from previous research and the potential for generating thousands of new markers from the two rice genome sequences [7, 130]. This has already had a pronounced impact on plant breeding and thisimpact will undoubtedly continue in the future. In theory, the lack of polymorphism for target markers in breeding material should no longer be a problem as more and more “allele-specific marker kits” will be available or be custom-made, where required for an increasing number of traits. Marker kits will enable the precise selection of parental lines for the generation of new breeding populations and reliable selection of segregating progeny. As more and more genes are identified, the development of “functional markers” or “perfect” markers will be more common [4]. Since functional markers are the site that determines phenotype, they are thus the ultimate marker in a marker kit. Such markers have been used for *Xa21* with great success [108–110]. Rice functional markers were recently developed for betaine aldehyde dehydrogenase (*BAD2;* controlling fragrance) and *xa5* was developed for bacterial blight resistance [223, 224].

### 4.2. Obstacles that genomics research will not solve

#### 4.2.1. Cost of using DNA markers

Despite the enormous potential for developing and using markers in rice, the cost of genotyping is still a prohibitive barrier to the wider application of MAS. Even with the global importance of rice, many developing countries have limited research and development capability. Therefore, cost optimization of current genotyping protocols and the development of new cost-effective protocols should be a major priority for breeding research and especially the rice molecular breeding lab. These improvements might involve simple optimizations of current laboratory practices, adopting new more efficient methods, or developing new MAS strategies and schemes.

Collard and Mackill [8] stated that preliminary cost analysis of MAS at IRRI indicated great potential for reduction. They stated a cost of US $1.00 per marker data point achieved by a post-doctoral research fellow or US $0.30 for a research technician, which we have since revised to US $0.37. At first glance, this amount may not sound like much, but when one considers that this indicates a cost of US $96 per plate, and that literally thousands (or even tens of thousands) of breeding lines are screened per annum in a typical rice breeding program, the importance of cost becomes obvious.

A detailed breakdown of cost components for the marker genotyping of a single SSR marker using standard methods indicated some interesting findings (Table 2).

- PCR costs the most in terms of consumables.
- The DNA extraction step costs the most in terms of labor.
- Overall, the DNA extraction step is the most expensive.

This analysis also provided a simple framework to investigate opportunities for some cost reduction (Table 2). In summary, scenarios 1 and 2 highlight that the optimization of technical procedures could decrease costs, scenario 3 highlights that the MAS scheme used will also vary costs, and scenario 4 shows that MAS lab planning and appropriate delegation of duties can also reduce costs.

Detailed cost-benefit analyses of using markers for specific traits could be critical information to determine the most appropriate and advantageous situations for using markers. For example, in maize, an extremely detailed cost-benefit analysis indicated that using markers for selection for *opaque2* (the gene associated with quality protein maize) was more economical than conventional screening methods [225]. In such cases, there is a clear-cut advantage of using markers in breeding.

#### 4.2.2. QTL application research: bridging the “application gap”

Many research steps are required from QTL discovery to the practical application of markers in a breeding program [69]. The three main research areas can be described as “QTL confirmation,” “broad-range QTL testing,” and “marker validation,” which we collectively refer to as “QTL application research.” These research areas have been loosely defined as QTL or marker validation activities—especially in wheat and barley. See references cited in [8]. However, in this paper, we have specifically defined the overall research area as QTL application research and have defined three components. QTL confirmation is desirable because factors such as small population sizes and insufficient replication of trait data, and experimental errors can cause inaccuracies in determining QTL positions and effects. Broad-range QTL testing refers to verification of QTLs in different populations by using previously reported markers in order to evaluate the effectiveness of the markers in predicting phenotype. This is required because of the effects of genetic background, possible epistatic interactions, and environmental effects that could ultimately reveal that QTLs may not be relevant in a specific breeding program. Marker validation activities are also required to evaluate the reliability of the markers and to identify polymorphism in relevant breeding lines. The latter two steps are also highly desirable for confirming marker-trait linkages identified by association mapping.

In practice, these research steps are often not performed and they represent an important obstacle for MAS to have an impact on crop improvement; this was referred to as the “application gap” by Collard and Mackill [8]. Although there are encouraging examples of marker validation research, there are relatively few published reports of QTL confirmation or broad-range application research in rice. A notable exception was the confirmation of QTLs for sheath blight resistance [226].

#### 4.2.3. “Phenotype gap”

This term was used to refer to the increasing ratio of genomic sequence data to known gene phenotype [227]—the term “phenotype gap” was originally coined by mammalian researchers. As mentioned earlier, this limits the ability to functionally annotate the constantly growing amount of rice genome sequence data. In the next few decades, the lack of knowledge of gene function will exist for the vast majority of rice genes. For mutant studies, only a few selected genotypes have been used, including only a single *indica* variety. Phenotypic analysis of mutant lines represents a considerable workload [142]. Precision phenotyping is also critical to the success of QTL or association mapping experiments, but, unfortunately, the importance of refining and developing new methods for precise phenotypic measurement is also often neglected in the genomics era. Overcomingthe phenotype gap represents the next great challenge for scientists involved in rice genomics research.

## 5. FUTURE CONSIDERATIONS FOR INTEGRATING THE RICE MOLECULAR BREEDING LABORATORY IN THE 21ST CENTURY

### 5.1. Molecular breeding lab activities

QTL application research activities represent an extensive amount of time, effort, and resources. In practice, it seems that molecular breeders will ultimately have to perform this research in situations, in which important data for the application of MAS are not available. From experience, it is clear that breeding programs that do not undertake these activities risk wasting considerable time and resources. However, in practice, QTL application research activities may be constrained by funding, time, and resources; in some cases, these activities may be beyond the capacity of many rice molecular breeding labs. Furthermore, a breeder may decide that, based on the importance of the target trait, such QTL application research steps do not worth the investment in time, resources, and money, since, at the end, the markers may not turn out to be useful for selection in their own breeding program.

This poses a practical barrier to the application of MAS in breeding programs for which there may not be any simple solutions. One possible solution that might assist plant breeders and molecular breeders could be the formation of molecular breeding networks in which practical information and experiences are readily shared between labs regarding specific gene/QTL targets and marker information. A web-based medium such as a “wiki” or electronic *Rice Molecular Breeding Newsletter* could be extremely useful. Greater integration with research objectives among the research institutes involved in QTL mapping might also result in more relevant data being generated for breeding programs.

Many activities will occur in the future rice molecular breeding lab. Obviously, the primary objectives will be to support and assist the breeding program in the evaluation and selection of breeding material. To fulfill this duty, organizational and maintenance activities such as organizing protocols, marker data, supplies of consumables, equipment maintenance, and LIMS will be critical. In-house data records for marker optimization and parental screening will be critical; generally, the more detailed the records, the better. This must include field and glasshouse leaf tissue collection protocols, which cannot be neglected.

It also seems certain that the development of custom-made markers will become more commonplace, and so molecular breeders will need to be proficient in skills such as PCR primer design, DNA sequence analysis, and using bioinformatics databases and tools. Considerable in silico applied genomics research will occur prior to wet-lab experiments or before breeding populations are initiated. SNPs will be the inevitable polymorphism target of choice arising from current and future genomics research, so rice molecular breeders should consider this ahead of time. Molecular breeders will also need to keep in touch with current bioinformatics tools and future genomics advances.

### 5.2. Integration within rice breeding programs

The advancements in the field of molecular breeding and genomics are proceeding at such a rapid rate that it makes it difficult for molecular breeders, let alone conventional plant breeders and other agricultural scientists, to keep abreast of these new developments. Thus, when possible, plant breeding stations that intend to adopt molecular breeding approaches should establish a molecular breeding lab with designated molecular breeders and technical staff, in order to maximize the likelihood of gaining benefits from molecular breeding. There will be a critical need for molecular breeders—like conventional plant breeders—to be Jacks (or Jills) of all trades in order to integrate the disciplines. In addition to a background in applied genomics, the ideal molecular breeder should have a strong background in classical and quantitative genetics and plant breeding. Molecular breeders will need to work extremely closely with senior plant breeders for trait prioritization and devising effective MAS strategies.

Of course, establishing molecular breeding labs will not be possible in many plant breeding stations, especially in developing countries, because of limited funding and resources. However, collaboration with national or international research institutions or universities could still provide opportunities for such breeding programs to gain benefits from genomics research.

For genomics to be fully integrated into the overall breeding program, we propose that molecular breeders be actively engaged in “genomics extension activities” (analogous to “agricultural extension”) to explain and disseminate information regarding markers and advances in genomics. Appropriate activities may include training workshops and developing practical manuals, booklets, and other educational material and would address the knowledge gap between molecular biologists, plant breeders, and other disciplines [8, 69]. Such activities might also encourage a greater integration in situations, in which university research labs conducting basic research are closely connected with actual breeding stations.

## 6. CONCLUDING REMARKS

Breeding research in rice is poised to gain many direct and indirect benefits from genomics research. However, there are many challenges for rice scientists to fully exploit and apply knowledge, resources, and tools in actual rice breeding programs. There are great opportunities for more efficient rice breeding and the faster development of new rice varieties in the future. We hope that some of the ideas proposed in this article will encourage the rice scientific community to collectively work toward converting rice from a model crop species into a model species for marker-assisted breeding.

## Figures and Tables

**Table 1:** Examples of marker-assisted selection in rice. na = not applicable.

Application	Traits or germplasm	Gene/QTLs	Markers used	Reference
Early generation selection	Bacterial blight	*Xa21*	STS	[78]
Gene surveys in parental material	Blast disease, predom. Korean germplasm	*Pi5(t)*	PCR/DNA gel blot	[79]
Gene surveys in parental material	Blast disease	*Pi-z*	SSR	[42]
Gene surveys in parental material	Blast disease	*Pi-ta*	Gene-specific marker	[80]
Genetic diversity assessment	Japonica varieties for hybrid combinations	na	SSR and RAPD	[77]
Genetic diversity assessment	Indian aromatic and quality rice	na	SSR	[74]
Genetic diversity assessment	U.S. varieties	na	SSR	[76]
Genetic diversity assessment	Nepalese landraces	na	SSRs	[81]
Genetic diversity assessment	Representative wild rice in China, *Oryza rufipogon* Griff.	na	SSR	[82]
Genetic diversity assessment	Indonesian varieties and landraces	na	SSR	[83]
Genotype identity testing	Hybrid rice	na	STS and SSR	[70, 72]
MABC	Bacterial blight	*Xa21*	STS and RFLP	[84]
MABC	Bacterial blight	*Xa21*	STS and AFLP	[85]
MABC	Bacterial blight	*xa5*	STS	[86]
MABC	Deep roots	QTLs on chromosomes 1, 2, 7, and 9	RFLP and SSR	[87]
MABC	Bacterial blight	*xa5, xa13, Xa21*	STS	[88, 89]
MABC	Blast	*Pi1*	SSR and ISSR	[90]
MABC	Quality	*Waxy*	RFLP and AFLP	[91]
MABC	Bacterial blight + quality	*xa13, Xa21*	STS, SSR, and AFLP	[92]
MABC	Submergence tolerance, disease resistance, quality	*Subchr9* QTL, *Xa21*, *Bph* and blast QTLs, and quality loci	SSR and STS	[93]
MABC	Blast disease	na	SSR	[94]
MABC	Root traits and aroma	QTLs on chromosomes 2, 7, 8, 9, and 11	RFLP and SSR	[95, 96]
MABC	Heading date	QTLs for heading date (*Hd1*, *Hd4*, *Hd5*, or *Hd6*)	RFLP, STS, SSR, CAPS, dCAPs	[97]
MABC	Submergence tolerance	*Sub1* QTL	SSR	[98]
MES	Indirect selection for adaptation	na	SSRs	[99]
Pyramiding	Bacterial blight	*Xa4, xa5, Xa10*	RFLP and RAPD	[100]
Pyramiding	Bacterial blight	*xa5, xa13, Xa4, Xa21*	RFLP, STS	[17]
Pyramiding	Blast disease	*Pi1, Piz-5, Pi2, Pita*	RFLP, STS	[101]
Pyramiding	Bacterial blight	*xa5, xa13*, *Xa21*	STS and CAPS	[102]
Pyramiding	Bacterial blight	*xa5, xa13, Xa21*	SSR and STS	[103]
Pyramiding	Bacterial blight and waxy genes	*xa5, xa13, Xa21, Wx*	SSR, STS, and CAPS	[104]
Pyramiding	Insect resistance and bacterial blight	*Xa21* and *Bt*	STS	[59]
Pyramiding	Brown plant-hopper	*Bph1* and *Bph2*	STS	[105]
Pyramiding	Thermosensitive genetic male sterility (TGMS) genes	*tms2, tgms,* *tms5*	SSR	[106]
Pyramiding	Bacterial blight	*Xa7* and *Xa21*	STS	[107]
Pyramiding	Bacterial blight	*Xa4, xa5,* and *Xa21*	STS	[108]
Pyramiding	Bacterial blight	*Xa4, Xa7,* and *Xa21*	STS	[109, 110]
Pyramiding/transgene selection	Blast and bacterial blight	*Pi-z and Xa21*	STS	[111]
Pyramiding/transgene selection	Bacterial blight	*xa5, xa13, Xa21*	STS (check)	[112]
Pyramiding/transgene selection	Bacterial blight, yellow stem borer, sheath blight	*Xa21, Bt RC7* chitinase gene, *Bt*	STS	[113]

**Table 2:** Cost breakdown of standard marker genotyping and exploration of marker genotyping cost reduction opportunities.

Situation	Step	Consumables (US $)	Labor (US $)	Cost per marker (US $)
Standard cost	DNA	0.051	0.437	**1.001**
	PCR	0.211	0.076	
	Gel	0.052	0.174	

Scenario 1—multiplex loading	DNA	0.051	0.437	**0.910**
	PCR	0.211	0.076	
	Gel	0.000	0.043	

Scenario 2—multiplex PCR	DNA	0.051	0.437	**0.500**
	PCR	0.211	0.076	
	Gel	0.052	0.174	

Scenario 3—MAS pyramiding	DNA	0.051	0.437	**0.676**
	PCR 1	0.211	0.076	
	Gel 1	0.052	0.174	
	PCR 2	0.211	0.076	
	Gel 2	0.052	0.174	
	PCR 3	0.211	0.076	
	Gel 3	0.052	0.174	

Scenario 4—DNA extraction performed by research technician	DNA	0.051	0.040	**0.604**
	PCR	0.211	0.076	
	Gel	0.052	0.174	

(i) Standard cost calculated based on the genotyping of 96 samples using a single SSR marker at IRRI from Collard and Mackill [8]. (ii) Data in this section of the table are reported for the second marker; hence, the gel cost for consumables is zero. The calculation was performed using the data for a standard marker plus the second marker (gel consumable cost = 0) and dividing by two. (iii) Multiplex loading by sequential loading of PCR samples, assuming different DNA samples are run in all lanes. Labor would require an extra 20 minutes for sequential loading, but gel preparation and assembly are no longer required in this scenario. (iv) If direct pooling of PCR products is possible, only a single loading is required for 96 samples (extra 5 minutes of labor). Gel labor costs are reduced to US $0.011 and the total cost per marker is $0.893. (v) Multiplex PCR (i.e., duplex PCR) in which two markers can be genotyped in the time and effort required for a single marker. (vi) For MAS pyramiding, the genotyping of three loci was considered. For simplicity, it was assumed markers could not be multiloaded, but obviously, if this was possible, it would indicate a further cost reduction per marker screened. (vii) The DNA extraction step is the most costly from our data analysis. There are considerable savings in expense, if genotyping efforts of a postdoctoral researcher are coordinated with those of a research technician at IRRI.

## ABBREVIATIONS

AFLP: amplified fragment length polymorphism;
BAC: bacterial artifical chromosome;
BC: backcross;
CAPS: cleaved amplified polymorphic site;
CG: candidate gene;
EST: expressed sequence tag;
FRET: fluorescence resonance energy transfer;
IL: introgression line;
ILP: intron length polymorphism;
ISSR: Inter-simple sequence repeats;
LIMS: laboratory information management system;
MABC: marker-assisted backcrossing;
MAS: marker-assisted selection;
MES: marker-evaluated selection;
NIL: near-isogenic lines;
PAC: P1 phage artificial chromosome;
PCR: polymerase chain reaction;
QTL: quantitative trait loci;
RAPD: random amplified polymorphic DNA;
RFLP: restriction fragment length polymorphism;
SBE: single base extension;
SCAR: sequence characterized amplified region;
SFP: single-feature polymorphism;
SNP: single nucleotide polymorphism;
SNuPE: single nucleotide primer extension;
SSCP: single-strand conformation polymorphism;
SSR: simple sequence repeats (microsatellites);
STS: sequence tagged site.
